# Cerium(III) Nitrate Containing Electrospun Wound Dressing for Mitigating Burn Severity

**DOI:** 10.3390/polym13183174

**Published:** 2021-09-18

**Authors:** Cortes Williams, Ramanda Chambers-Wilson, Jahnabi Roy, Christine Kowalczewski, Angela R. Jockheck-Clark, Robert Christy, Luis A. Martinez

**Affiliations:** 1 Naval Medical Research Unit San Antonio, JBSA-Fort Sam Houston, San Antonio, TX 78234, USA; ramanda.t.chamberswilson.ctr@mail.mil (R.C.-W.); luis.a.martinez298.civ@mail.mil (L.A.M.); 2 United States Army Institute of Surgical Research, JBSA-Fort Sam Houston, San Antonio, TX 78234, USA; jahnabi.roy.ctr@mail.mil (J.R.); christine.j.kowalczewski.ctr@mail.mil (C.K.); angela.r.jockheck-clark.ctr@mail.mil (A.R.J.-C.); robert.j.christy12.civ@mail.mil (R.C.)

**Keywords:** electrospinning, burn dressing, wound dressing, cerium nitrate, cerium(III) nitrate, nanoencapsulation, nanofibers

## Abstract

Thermal injuries pose a risk for service members in prolonged field care (PFC) situations or to civilians in levels of lower care. Without access to prompt surgical intervention and treatment, potentially salvageable tissues are compromised, resulting in increases in both wound size and depth. Immediate debridement of necrotic tissue enhances survivability and mitigates the risks of burn shock, multiple organ failure, and infection. However, due to the difficulty of surgical removal of the burn eschar in PFC situations and lower levels of care, it is of utmost importance to develop alternative methods for burn stabilization. Studies have indicated that cerium(III) nitrate may be used to prolong the time before surgical intervention is required. The objective of this study was to incorporate cerium(III) nitrate into an electrospun dressing that could provide burst release. Select dosages of cerium(III) nitrate were dissolved with either pure solvent or polyethylene oxide (PEO) for coaxial or traditional electrospinning set-ups, respectively. The solutions were coaxially electrospun onto a rotating mandrel, resulting in a combined nonwoven mesh, and then compared to traditionally spun solutions. Dressings were evaluated for topography, morphology, and porosity using scanning electron microscopy and helium pycnometry. Additionally, cerium(III) loading efficiency, release rates, and cytocompatibility were evaluated in both static and dynamic environments. Imaging showed randomly aligned polymer nanofibers with fiber diameters of 1161 ± 210 nm and 1090 ± 250 nm for traditionally and coaxially spun PEO/cerium(III) nitrate dressings, respectively. Assay results indicated that the electrospun dressings contained cerium(III) nitrate properties, with the coaxially spun dressings containing 33% more cerium(III) nitrate than their traditionally spun counterparts. Finally, release studies revealed that PEO-based dressings released the entirety of their contents within the first hour with no detrimental cytocompatibility effects for coaxially-spun dressings. The study herein shows the successful incorporation of cerium(III) nitrate into an electrospun dressing.

## 1. Introduction

Thermal burns that require medical intervention affect nearly 500,000 people and account for approximately 3400 deaths annually in the US [1]. Patients who have received major burn damage, total burn surface area (TBSA) > 20%, typically undergo care at a specialized burn center, with a survival rate of above 97% [2]. However, in cases where immediate transport to a burn center is not possible, or for burns resulting from military conflict, survival rates drop as low as 80% [3,4]. In these situations, the removal of necrotic tissue and access to IV fluid resuscitation may be delayed for more than 72 h, which increases the chances of sepsis, burn shock, multiple organ failure, and mortality [5]. To combat the aforementioned issues and minimize the burn morbidities associated with prolonged field care scenarios, novel wound dressings are needed to help extend the time needed for patients to receive specialized care.

The severity of a thermal burn is classified by its depth, and it is generally accepted that thermal burns consist of three zones that vary in both the extent and depth of tissue damage [1,5,6]. The zone of necrosis (coagulation) is the area that sustains the greatest damage from the thermal trauma and suffers irreversible destruction of its vascular system. It is characterized by dead cells and necrotic tissue that is collectively known as the burn eschar. The zone of hyperemia, conversely, will eventually recover without additional treatment. The middle area, the zone of stasis, contains highly stressed tissue that, without intervention, will die and allow for wound progression. As such, it is of vital importance to focus burn care on cultivating the tissue in each of these zones, especially in prolonged field care scenarios [1].

The gold standard for burn treatment is immediate debridement, or surgical removal, of the dead tissue [1]. Unfortunately, in extended field situations, surgical debridement methods are not readily available [3]. For the burn eschar, in particular, the dying cells leach toxic metabolites that can have detrimental effects on the surrounding tissue, and studies have shown that delayed grafting leads to reduced survivability [5,6,7,8].

Topical treatment of the burn area with cerium(III) nitrate (Ce(III)), either by itself or in combination with silver sulfadiazine, has been shown to delay the need for debridement [7,8,9,10,11]. Burn eschars treated with Ce(III) become firm and leather-like, slowing the leaching of toxic metabolites but do not separate from the wound area. However, once excised, the tissue beneath the eschar is generally healthy and has a high rate (>90%) of graft acceptance. Additionally, multiple studies have shown Ce(III) may maintain late-stage burn immune responses and reduce levels of immunomodulatory cytokines by binding and denaturing the lipid-protein complex. For delivery, Ce(III) has been used in both commercially available creams and incorporated into gelatin- and chitosan-based film dressings [8,9,10,12]. Creams and hydrogels, unfortunately, are not suitable for prolonged field care use due to the amount of space they require in a medic’s pack. An alternative delivery method would be the incorporation of Ce(III) in a nonwoven fiber dressing resembling traditional gauze [13,14,15].

Nanomaterials are increasingly becoming more popular for use as wound dressings due to their high surface area-to-volume ratios, variable degradation rates, and ability for controlled drug delivery [16,17,18,19,20,21,22,23,24,25]. Additionally, the nonwoven fibers fabricated through electrospinning exhibit fiber diameters on the nanoscale (<1 µm) range, which is consistent with collagen fibril diameters in the extracellular matrix (<500 nm). Furthermore, electrospinning fabrication techniques are compatible with a wide range of polymers, both natural and synthetic, allowing researchers to pick the polymer with the most optimal properties for the application. Due to these characteristics, electrospinning is at the forefront for fabricating functionalized nanomaterial scaffolds for burn wound dressing applications.

Electrospinning also allows for the use of various protective techniques in order to not only retain the moiety bioactivity but also reduce environmental effects on that moiety. By altering various conditions such as voltage, flight distance, solvent systems, and solvent conductivity, the resulting fibers may express a wide range of fiber diameters and dressing porosities. Nanoparticles are most commonly used in conjunction with electrospinning in order to control drug release profiles [19,20,24,25,26,27]. Alternately, coaxial electrospinning can be used to encase a sensitive moiety “core” within a protective polymer “shell” [16,18,27,28,29,30,31,32]. This method traditionally consists of electrospinning one polymer inside of another resulting in a core-shell arrangement, with the drug contained within the core.

In order to combat the aforementioned issues associated with the treatment of burn pathologies in areas without a burn center, the objective of this study was to incorporate Ce(III) into an electrospun dressing that could provide a burst release of treatment. The polymer vehicle for the dressing was polyethylene oxide, which is known for having a very rapid degradation rate, rapid dissolution in physiological environments, and high biocompatibility. In addition, a modified coaxial-electrospinning method was utilized to encapsulate cerium within the fibers while simultaneously stopping hygroscopic effects, which is the Ce(III) tendency to uptake water from the atmosphere resulting in oxidation. The resulting nanofibers were characterized and evaluated for their biocompatibility, Ce(III) loading capacity, and release kinetics using in vitro methods.

## 2. Materials and Methods

### 2.1. Materials

Acetone (97%), polyethylene oxide (PEO; MW = 300,000), cerium(III) nitrate hexahydrate (Ce(III)), phosphate-buffered saline, sodium triphosphate, Tris-buffered saline (TBS), and dichloromethane (DCM) were purchased from Sigma-Aldrich (St. Louis, MO, USA). Human dermal fibroblasts (PCS-201-012) and cell culture reagents were purchased from American Type Culture Collection (ATCC; Manassas, VA, USA). CyQUANT^®^ cell proliferation assay kits were purchased from Thermo Fisher Scientific (Waltham, MA, USA).

### 2.2. Preparation of Electrospun Cerium(III) Nitrate Containing Polyethylene Oxide Dressings

**Figure 1:**
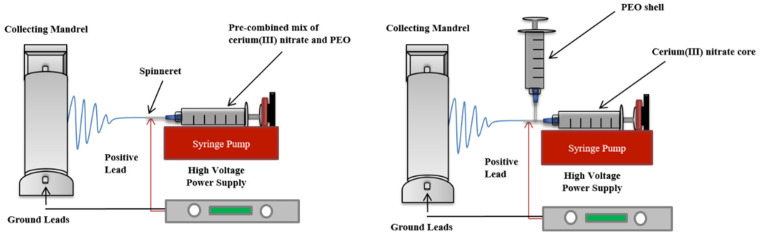
Electrospinning process orientations. Traditional spinning with one solution (**left**) and coaxial encapsulation (**right**).

Solutions containing PEO and Ce(III) were made using three different methods, corresponding to the electrospinning technique used to fabricate the nonwoven mesh dressing. The electrospinning set-ups utilized are shown in Figure 1, above.

For use in traditional electrospinning, PEO and Ce(III) were dissolved at a mass ratio of 1:1 in a 2:1 (*v*/*v*) ratio of acetone to DCM to obtain a total polymer content of 5% *w*/*v*, which has been shown to be a very stable solvent system for PEO. The resulting combined solution was loaded onto a syringe pump (NE-1000, New Era Pump Systems, NY, USA). The solution was electrospun through an 18 gauge spinneret, with a flight distance of 15 cm and relative humidity of 10%. Supplied voltage ranged from 10–18 kV, and the associated flow rate ranged from 1.05–5 mL/h. Fibers were collected on a grounded mandrel rotating at 25 rpm.

For dressings fabricated using the coaxial set-up, PEO solutions were made by following the steps stated above. A separate solution of Ce(III) was made by dissolving 5% *w*/*v* Ce(III) in acetone. Following this, each solution was loaded onto a separate syringe pump connected to a different inlet of an 18/16 gauge coaxial needle, and flow rates were initially set to 1.5 mL/h and 0.15 mL/h for PEO and Ce(III) solutions, respectively, and slowly increased to 5 mL/h and 0.5 mL/h for PEO and Ce(III) solutions, respectively. For these samples, variability was mitigated by electrospinning the same volume of polymers for each batch. The solutions were spun at 28 kV with a flight distance of 12 cm onto a mandrel rotating at 25 rpm.

### 2.3. Dressing Characterization

Polymer electrospinning solutions were evaluated for conductivity using a Malvern Zetasizer Nano (Malvern Panalytical, Roystone, UK), following manufacturer protocols. Electrospun dressings were analyzed for average pore size and fiber diameter using field emission scanning electron microscopy (SEM) (Zeiss Sigma VP-40, Carl Zeiss AG, Jena, Germany) following spatter-coating with gold palladium, and porosity using helium pycnometry. Three dressings were analyzed per dressing composition, with 30 fibers and 30 pores evaluated for each, using ImageJ (NIH, Bethesda, MD, USA). Additionally, dressings were analyzed using Fourier Transform Infrared Spectroscopy (FTIR, Spectrum 400 ATR-FTIR PerkinElmer, Waltham, MA, USA) at a range of 4000–500 cm^−1^ to confirm the presence of Ce(III) [14].

### 2.4. In Vitro Dressing Degradation and Loading Potential

Dressing degradation was determined in deionized water at room temperature. Ce(III) has been shown to interact negatively with phosphate-based buffers and cell culture medium [13,33]. Scaffolds (n = 3) were immersed in 10 mL of deionized (DI) water and incubated for up to 3 h. Following complete degradation, Ce(III) concentrations of samples were measured using a rapid analytical fluorometric detection method that was developed to determine trace amounts of Ce(III) in aqueous samples [34,35]. Additionally, samples were taken and weighed from various locations of spun dressings in order to test batch to batch variability.

### 2.5. Cerium(III) Release Rate

The amount of Ce(III) released over time by the electrospun dressings was evaluated using a PermeGear^®^ in-line diffusion system (Twin-Flow, Dual In-Line; Hellertown, PA, USA). In brief, a total of 9 Ce(III) containing electrospun wound dressings were cut to a diameter of 25 mm and applied to a Durapore^®^ hydrophilic polyvinylidene fluoride (PVDF, EMD Millipore, MA, USA) 0.45 µm filter. The wound dressings and filter were placed over a support in the PermeGear^®^ in-line flow-through diffusion cell, pre-wet with TBS, and clamped into position. The inlet port of the diffusion cell was attached to a multichannel peristaltic pump (NE-1600, New Era Pump System, NY, USA), and TBS was continuously pumped through each cell at a flow rate of 10–20 µL/min at 37 °C. Samples were collected at 1, 3, and 16 h through the in-line diffusion cell outlet port using an automated fraction collector and measured using the aforementioned fluorometric detection assay.

### 2.6. In Vitro Cell Cytotoxicity

Cytotoxicity of the dressings was evaluated using CyQUANT^®^ assay kits following manufacturer-recommended methods. Human dermal fibroblasts were cultured to 80% confluency, trypsinized, and re-suspended at a concentration of 10,000 cells/mL of media, and seeded into 6-well plates. After a 24-h seeding period, media was refreshed, and the study groups (no treatment control, PEO scaffold control, pure Ce(III) control, coaxially spun scaffold, and dual-spun scaffold) were placed directly into the wells (n = 3). At 24 h, dressings were removed, and cells were briefly washed with buffer and cultured for an additional 48 h. This resulted in a 72-h culture period with a 24-h treatment.

### 2.7. Statistical Analyses

Statistical analyses were conducted using GraphPad Prism (San Diego, CA, USA). Quantitative data is represented as the mean ± standard deviation and was calculated using analysis of variance (ANOVA) with a posthoc Tukey test. *p* < 0.05 was considered statistically significant.

## 3. Results and Discussion

### 3.1. Properties of PEO/Ce(III) Solutions

Control PEO, Ce(III) nitrate, and PEO/Ce(III) nitrate solutions were evaluated for conductivity (Table 1). PEO in acetone, DCM solvent is widely considered one of the most stable solutions for electrospinning [19,34,35], and this is supported by the observed low conductivity shown in Table 1. Conversely, Ce(III) had a relatively high conductivity. This was observed during the electrospinning process by its difficulty incorporating into polymer fibers, resulting in a wide range of fiber diameters, as seen in Table 2. Also of note, the addition of Ce(III) with PEO in acetone, DCM solutions causes the PEO to drop out of solution.

### 3.2. Morphology and Properties of PEO/Ce(III) Nanofibers

**Figure 2:**
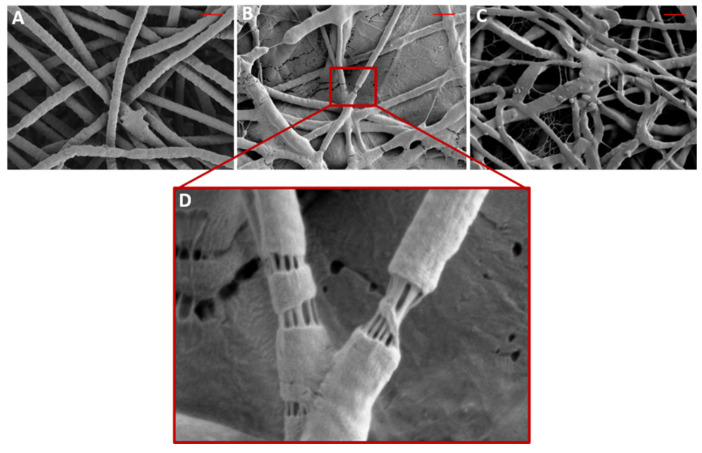
Representative SEM images of (**A**) electrospun PEO, (**B**) dual-spun PEO/Cerium(III) nitrate, (**C**) coaxially electrospun PEO/Cerium(III) nitrate scaffolds, (**D**) core-shell formation in coaxially electrospun PEO/Cerium(III) nitrate. Scale bar = 4 µm; Red area = 64 µm^2.^

SEM micrographs were taken of electrospun control PEO, dual-spun PEO/Ce(III) nanofibers, and coaxial-spun nanofibers (Figure 2) and evaluated for fiber diameter and membrane porosity (Table 2). Surprisingly, loaded scaffolds had the smallest mean fiber diameter, not normally seen in the literature [16,18,28,30,31,32]. Additionally, these groups had statistically significant lower mean dressing porosities, 46.5% ± 2.6 for coaxial-spun, and 49.0% ± 1.7 for dual-spun. Dressing porosity and fiber diameter can have a high impact on dressing dissolution rate, degradation, and breathability [19,34,35]. Most importantly, SEM imaging confirmed the presence of the PEO/Ce(III) core-shell architecture, the importance of which will be discussed in Section 3.3. The magnified inset in Figure 2 shows a PEO polymer shell that has been stretched open to show its interior.

### 3.3. Dressing Chemical Composition

**Figure 3:**
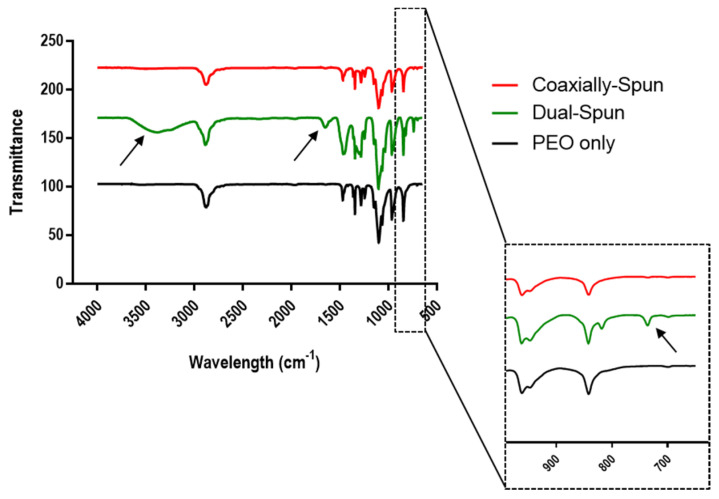
FTIR spectra of electrospun PEO (black), dual-spun PEO/Cerium(III) nitrate (green), and coaxially-spun PEO/Cerium(III) nitrate (red) scaffolds. Arrows indicate known peaks (3387 and 1641 are O-H groups of bound moisture, and ~700 is Ce(III)) [14].

The chemical composition of the electrospun samples was evaluated using FTIR, shown in Figure 3. The arrows point to various peaks specific to Ce(III). The peak seen just above 700 cm^−1^ indicates the presence of Ce(III), while the peaks at 3387 cm^−1^ and 1641 cm^−1^ are O-H groups from bound moisture [13]. Bound moisture is present on FTIR spectra due to the hygroscopic characteristics of Ce(III). This moisture is not present in the coaxially-spun samples, further supporting the success of encapsulation.

### 3.4. Cerium(III) Nitrate Loading Potential

**Figure 4:**
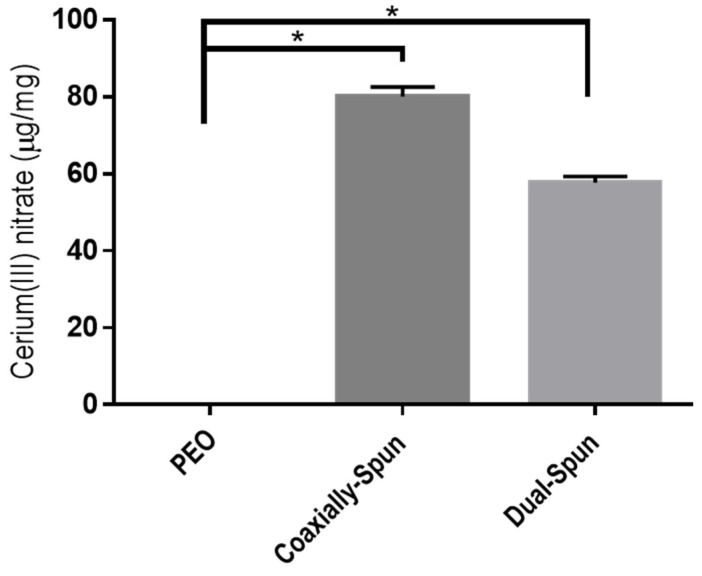
Loading potential of electrospun dressings (n = 3). * denotes a statistically significant difference (*p* < 0.05).

The loading potential, amount of Ce(III) contained within each electrospun dressing, was evaluated on each dressing type. After drying, dressings were weighed then dissolved in TBS; results were then compared on a per weight basis. Figure 4 shows the resulting loading capacity across various electrospinning batches. Ce(III) loading capacities average 80 µg/mg for coaxially spun dressings and just shy of 60 µg/mg for dual-spun dressings. The lower amount of Ce(III) detected in the dual-spun samples may be due to the interactions between Ce(III) and PEO. First, Ce(III) has a very high conductivity, which causes it to carry the electrical charge throughout the electrospinning process, drying the solution [36,37]. This is coupled with its limited solubility in PEO. The less polymer in solution would then result in less polymer to carry Ce(III) across the air gap and less Ce(III) in the final fiber mat. Coaxial set-ups solve this issue by keeping the Ce(III) in the interior of the fibers and thereby mitigating these undesirable interactions.

### 3.5. Cerium(III) Release Rate

**Figure 5:**
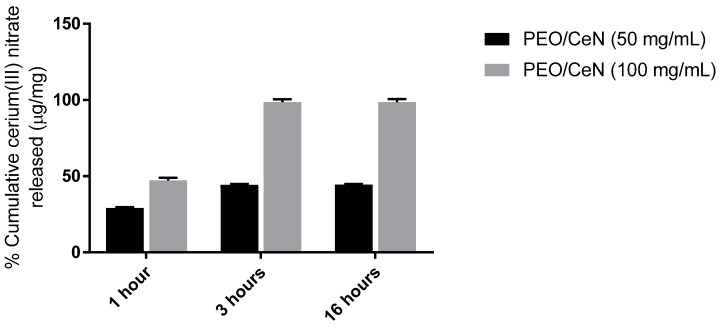
% Cumulative Ce(III) released per scaffold (µg/mg) over 16 h for 50 mg_scaffold_/mL_diluent_ and 200 mg_scaffold_/mL_diluent_ PEO/Ce(III) (n = 9).

Cumulative release of Ce(III) was demonstrated in samples collected from coaxially spun, cerium(III) nitrate-containing scaffolds using the PermeGear^®^ ILC-07 automated system. The results of the in vitro diffusion study demonstrated a burst release of Ce(III) at 1 h. The amount of Ce(III) collected decreased at 3 h, and only trace amounts of Ce(III) were observed at 16 h. Additionally, there is dose-dependent release control, seen by the increase in overall release amounts for dressings with a higher initial loading (Figure 5).

### 3.6. Viability of Human Dermal Fibroblasts

**Figure 6:**
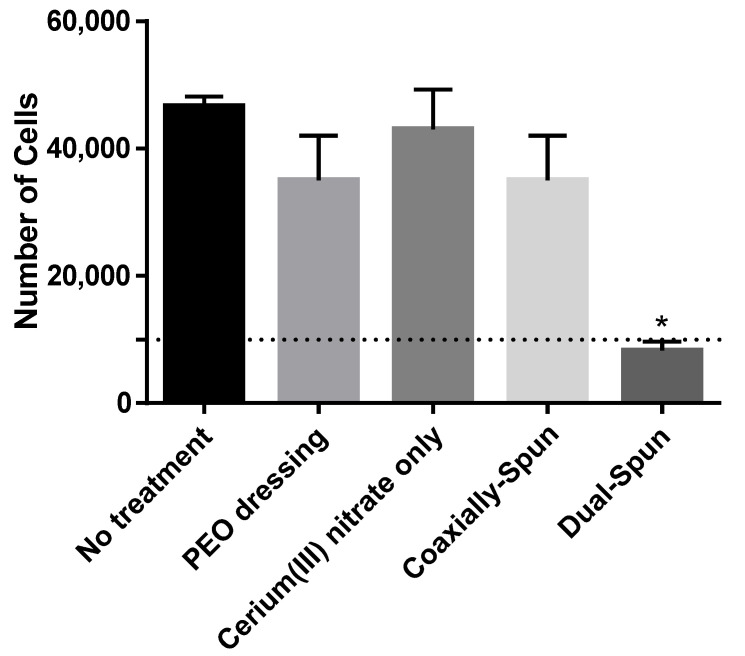
Cell proliferation of human dermal fibroblasts over 72 h (n = 3). Dashed line indicates the original seeding density. * denotes a statistically significant difference (*p* < 0.05).

Viability of primary human fibroblasts was performed on each dressing type and compared against PEO control dressing and Whatman^®^ paper soaked in 5% (*w*/*v*) Ce(III) in acetone (Figure 6). When comparing the controls, there are no significant differences in cell viability. Over 72 h, there are no significant differences between the controls and the coaxially-spun samples; however, the dual-spun group shows a statistically significant decrease in proliferation (possibly due to the exposed Ce(III), as discussed previously in Section 3.3). The exposure of Ce(III) to the atmosphere, and its subsequent oxidation prior to cell seeding, seem to play a role in the dressing’s decreased cytocompatibility in comparison to coaxially-spun counterpart.

## 4. Conclusions

In this work, Ce(III) was encapsulated within electrospun polyethylene oxide nanofibers and characterized for its potential use as a treatment containing burn dressing. Results shown herein indicated that coaxial electrospinning was capable of containing Ce(III) within the interior of the nanofibers while also shielding the Ce(III) from hygroscopic effects. Additionally, this finding was supported in in vitro loading capacity studies, wherein coaxially spun dressings contained statistically more Ce(III) than the traditionally dual spun dressings. Although the dual-spun Ce(III)-containing dressings experienced decreased cell proliferation in in vitro tests, coaxially-spun dressings performed statistically equivalent to the control groups. Finally, coaxially spun dressings were capable of delivering a burst release of Ce(III). In conclusion, the work presented demonstrates the applicability of coaxial electrospinning for the encapsulation and subsequent fabrication of difficult-to-handle moieties without the use of a secondary polymer vehicle. Further, this study lays the groundwork for the development of Ce(III) dressings for thermal injury treatment and its use as a point-of-injury treatment for thermal injuries and a potential means of mitigating the deleterious effect of delayed surgical debridement.

## Figures and Tables

**Table 1:** Conductivity of PEO/Cerium(III) nitrate solutions.

Sample	Conductivity ± SD (ms/cm) *
5% *w*/*v* PEO in 1:1 acetone, DCM	0.00399 ± 0.000915
5% *w*/*v* Ce(III) nitrate in acetone	2.12 ± 0.114
5% *w*/*v* PEO in 1:1 acetone, DCM + 5% *w*/*v* Ce(III) nitrate in acetone	0.132 ± 0.00651

(*) all values significant.

**Table 2:** Fiber diameter and membrane porosity of electrospun scaffolds.

Sample	Average Fiber Diameter ± SD (µm)	Average Porosity (%)
PEO, control	1.80 ± 0.10 *	56.6 ± 0.5 *
Dual-spun PEO/Ce(III)	1.16 ± 0.21	49.0 ± 1.7
Coaxially-spun PEO/Ce(III)	1.09 ± 0.25	46.5 ± 2.6

(*) denotes significance.

## Data Availability

Data available on request due to restrictions.

## References

[1] Rowan M.P., Cancio L.C., Elster E.A., Burmeister D.M., Rose L.F., Natesan S., Chan R.K., Christy R.J., Chung K.K. (2015). Burn wound healing and treatment: Review and advancements. Crit. Care.

[2] Pavoni V., Gianesello L., Paparella L., Buoninsegni L.T., Barboni E. (2010). Outcome predictors and quality of life of severe burn patients admitted to intensive care unit. Scand. J. Trauma Resusc. Emerg. Med..

[3] Chan R.K., Aden J., Wu J., Hale R.G., Renz E.M., Wolf S. (2015). Operative Utilization Following Severe Combat-Related Burns. J. Burn. Care Res..

[4] Kauvar D.S., Wolf S.E., Wade C.E., Cancio L.C., Renz E.M., Holcomb J.B. (2006). Burns sustained in combat explosions in Operations Iraqi and Enduring Freedom (OIF/OEF explosion burns). Burns.

[5] Schmauss D., Rezaeian F., Finck T., Machens H.-G., Wettstein R., Harder Y. (2015). Treatment of Secondary Burn Wound Progression in Contact Burns—A Systematic Review of Experimental Approaches. J. Burn. Care Res..

[6] Bertin-Maghit M., Goudable J., Dalmas E., Steghens J.P., Bouchard C., Gueugniaud P.Y., Petit P., Delafosse B. (2000). Time course of oxidative stress after major burns. Intensive Care Med..

[7] Deveci M., Eski M., Sengezer M., Kisa U. (2000). Effects of cerium nitrate bathing and prompt burn wound excision on IL-6 and TNF-α levels in burned rats. Burns.

[8] Scholten-Jaegers S.M.H.J., Nieuwenhuis M.K., Van Baar M.E., Niemeijer A.S., Hiddingh J., Beerthuizen G.I.J.M. (2017). Epidemiology and Outcome of Patients with Burns Treated with Cerium Nitrate Silversulfadiazine. J. Burn. Care Res..

[9] Garner J., Heppell P. (2005). Cerium nitrate in the management of burns. Burns.

[10] Oen I.M.M.H., Van Baar M.E., Middelkoop E., Nieuwenhuis M.K. (2012). Effectiveness of cerium nitrate–silver sulfadiazine in the treatment of facial burns: A multicenter, randomized, controlled trial. Plast. Reconstr. Surg..

[11] Scheidegger D., Sparkes B., Lüscher N., Schoenenberger G., Allgöwer M. (1992). Survival in major burn injuries treated by one bathing in cerium nitrate. Burns.

[12] Huang C., Huang Y., Tian N., Tong Y., Yin R. (2010). Preparation and characterization of gelatin/cerium(III) film. J. Rare Earths.

[13] Augustine R., Hasan A., Patan N.K., Dalvi Y.B., Varghese R., Antony A., Unni R.N., Sandhyarani N., Al Moustafa A.-E. (2020). Cerium Oxide Nanoparticle Incorporated Electrospun Poly(3-hydroxybutyrate-co-3-hydroxyvalerate) Membranes for Diabetic Wound Healing Applications. ACS Biomater. Sci. Eng..

[14] Babu S., Velez A., Wozniak K., Szydlowska J., Seal S. (2007). Electron paramagnetic study on radical scavenging properties of ceria nanoparticles. Chem. Phys. Lett..

[15] Kirkbright G., West T., Woodward C. (1966). Some spectrofluorimetric applications of the cerium(IV)-cerium(III) system. Anal. Chim. Acta.

[16] Alharbi H.F., Luqman M., Khalil K.A., Elnakady Y.A., Elkader O.A., Rady A.M., Alharthi N.H., Karim M.R. (2018). Fabrication of core-shell structured nanofibers of poly (lactic acid) and poly (vinyl alcohol) by coaxial electrospinning for tissue engineering. Eur. Polym. J..

[17] Chen S., Liu B., Carlson M.A., Gombart A.F., Reilly D.A., Xie J. (2017). Recent advances in electrospun nanofibers for wound healing. Nanomedicine.

[18] Chou S.-F., Carson D., Woodrow K.A. (2015). Current strategies for sustaining drug release from electrospun nanofibers. J. Control. Release.

[19] Hu X., Liu S., Zhou G., Huang Y., Xie Z., Jing X. (2014). Electrospinning of polymeric nanofibers for drug delivery applications. J. Control. Release.

[20] Maleki M., Amani-Tehran M., Latifi M., Mathur S. (2014). Drug release profile in core–shell nanofibrous structures: A study on Peppas equation and artificial neural network modeling. Comput. Methods Programs Biomed..

[21] Perez R., Kim H.-W. (2015). Core–shell designed scaffolds for drug delivery and tissue engineering. Acta Biomater..

[22] Rescignano N., Fortunati E., Montesano S., Emiliani C., Kenny J.M., Martino S., Armentano I. (2014). PVA bio-nanocomposites: A new take-off using cellulose nanocrystals and PLGA nanoparticles. Carbohydr. Polym..

[23] Yao C.-H., Lee C.-Y., Huang C.-H., Chen Y.-S., Chen K.-Y. (2017). Novel bilayer wound dressing based on electrospun gelatin/keratin nanofibrous mats for skin wound repair. Mater. Sci. Eng. C.

[24] Yoo H., Kim T.G., Park T.G. (2009). Surface-functionalized electrospun nanofibers for tissue engineering and drug delivery. Adv. Drug Deliv. Rev..

[25] Zhu T., Yang C., Chen S., Li W., Lou J., Wang J. (2015). A facile approach to prepare shell/core nanofibers for drug controlled release. Mater. Lett..

[26] Fredenberg S., Wahlgren M., Reslow M., Axelsson A. (2011). The mechanisms of drug release in poly(lactic-co-glycolic acid)-based drug delivery systems—A review. Int. J. Pharm..

[27] Tiwari S.K., Tzezana R., Zussman E., Venkatraman S.S., Venkatraman S. (2010). Optimizing partition-controlled drug release from electrospun core–shell fibers. Int. J. Pharm..

[28] Choi J.S., Choi S.H., Yoo H. (2011). Coaxial electrospun nanofibers for treatment of diabetic ulcers with binary release of multiple growth factors. J. Mater. Chem..

[29] Jiang H., Hu Y., Li Y., Zhao P., Zhu K., Chen W. (2005). A facile technique to prepare biodegradable coaxial electrospun nanofibers for controlled release of bioactive agents. J. Control. Release.

[30] Jiang H., Wang L., Zhu K. (2014). Coaxial electrospinning for encapsulation and controlled release of fragile water-soluble bioactive agents. J. Control. Release.

[31] Nguyen T.T.T., Ghosh C., Hwang S.-G., Chanunpanich N., Park J.S. (2012). Porous core/sheath composite nanofibers fabricated by coaxial electrospinning as a potential mat for drug release system. Int. J. Pharm..

[32] Nguyen T.T.T., Chung O.H., Park J.S. (2011). Coaxial electrospun poly(lactic acid)/chitosan (core/shell) composite nanofibers and their antibacterial activity. Carbohydr. Polym..

[33] Singh V., Singh S., Das S., Kumar A., Self W.T., Seal S. (2012). A facile synthesis of PLGA encapsulated cerium oxide nanoparticles: Release kinetics and biological activity. Nanoscale.

[34] Pakravan M., Heuzey M.-C., Ajji A. (2011). A fundamental study of chitosan/PEO electrospinning. Polymer.

[35] Xie J., Li X., Xia Y. (2008). Putting Electrospun Nanofibers to Work for Biomedical Research. Macromol. Rapid Commun..

[36] Angammana C.J., Jayaram S.H. (2011). Analysis of the Effects of Solution Conductivity on Electrospinning Process and Fiber Morphology. IEEE Trans. Ind. Appl..

[37] Uyar T., Besenbacher F. (2008). Electrospinning of uniform polystyrene fibers: The effect of solvent conductivity. Polymer.

